# Medical Wards of the Mercy Hospital

**Published:** 1864-10

**Authors:** N. S. Davis

**Affiliations:** Prof. of Practical and Clinical Medicine


### The Clinique.

### MEDICAL WARDS OF THE MERCY HOSPITAL.

### CHRONIC DIARRHŒA —— CAMP DIARRHŒA, &c.

BY N. S. DAVIS, M.D., Prof. of Practical and Clinical Medicine.

CASE I. This man, aged about 35 years, has recently returned from the army in the South. He was admitted into the hospital yesterday. I learn that he has had diarrhœa constantly for the last six or eight months. You see his skin is dark color, exceedingly rough and dry; he is greatly emaciated; countenance haggard; lips thin, pale, and dry; tongue and gums pale, but clean, and inclined to dryness; little or no appetite; abdominal walls sunken, but not tender; intestinal discharges frequent, generally large in quantity, thin, and of a pale yellow or ash-gray color, and accompanied by very little pain, though sometimes they have been small in quantity, mixed with mucus and tinged with blood. His urinary secretion is scanty, nearly natural in color when voided, but becomes turbid with phosphatic salts on standing. The feet and ankles are œdematous, especially when allowed to remain in a dependent position. His pulse is small, feeble, and about 100 per minute.

CASE II. Turning to the next bed we find another patient, also from the army. He was brought into the hospital, two days since, in a state of extreme exhaustion. His expression of face is strongly hippocratic; his tongue and gums are less pale than in the first case, but look dry, tender, and present some points of apthous ulceration. The skin is even more rough and dry than in the other case; the pulse is very feeble and quick; the extremities cool and extremely emaciated. His pulse is small, feeble, and frequent. He has had diarrhœa, sometimes assuming the character of dysintery, for several months. His discharges are at present dark colored, thin, and tinged with blood, and are sometimes passed involuntarily.

Case III. This man, aged about 30 years, has also been in the South during the past two years. He has not, however, the extreme emaciation or haggard countenance presented by the other two. His whole cutaneous surface is white or bloodless, while the lips, tongue and gums are remarkably pale. His skin is cool; pulse soft and only slightly increased in frequency; tongue clean, and appetite fair, though digestion is often accompanied by flatulency. He has been affected with a moderate chronic diarrhœa during the last four months. The discharges have been thin and copious, but usually not more than four or five in the twenty-four hours. Though able to be up, he is quickly exhausted by muscular exercise.

The three foregoing cases illustrate different varieties and stages of a disease that has destroyed the lives of a far larger number of our soldiers than all the armed rebels of the South. The change of diet, habits, and climate involved in the sudden transition from civil life in the North to the military camps in the South, causes a great prevalence of diarrhœa and dysentery, and many of the cases are protracted in a chronic form. From such of the latter as have come under my observation, I think the symptoms justify their arrangement into two classes. One embraces such cases as are characterized by some pain and soreness in the abdomen; a very dry, harsh skin; rapid emaciation; increased frequency of pulse; and frequent intestinal discharges, generally thin, redish yellow or dark brown, containing some mucus, and sometimes tinged with blood. The other class embraces such cases as present little pain or tenderness in the abdomen; little emaciation; a pulse nearly natural in frequency; and instead of a rough and dry skin, the whole cutaneous surface appears bloodless, with paleness of the lips and often a sallow hue of the face. The intestinal discharges are copious, very thin, and generally of a pale yellow or gray, ish color, and seldom contain any appearance of mucus. The third patient now before the class, is a fair sample of this variety of cases; while the first and second are specimens of the other class in an extreme stage of advancement. In the first class of cases there is evidently an inflammatory condition of the mucous membrane of the ilium or colon, or both. The altered properties of the membrane, and the accumulation of blood in it, constituting such inflammatory condition, is, not only productive of morbid secretion, as seen in the frequent and unnatural evacuations, but also of infiltration into the sub-mucous tissue, causing tumefaction and subsequently induration with abrasions and ulcerations upon the surface. In the second class of cases there is much less evidence of inflammatory action, the special pathological condition of the mucous membrane being that of extreme irritability, coupled with such a perversion of function in the capillaries, as to cause an almost constant effusion of fluids instead of absorption. This extreme sensitiveness sometimes extends throughout the whole alimentary canal, and even into the lining of the hepatic ducts. In such cases whatever food or drink is taken, is speedily followed by such excessive peristaltic movements that a large part of it is hurried through the intestines without digestion or absorption. Consequently the patient becomes rapidly anemic; weak; countenance sallow or of a waxy paleness; and the watery element of the blood so much in excess as to cause œdematous infiltrations into the feet and ankles, or whatever part may remain most dependent. In the treatment of this class of cases of chronic diarrhœa there are three plain indications to be fulfilled, namely: to allay the morbid sensibility of the mucous membrane and thereby quiet the excessive peristaltic movements; to correct the perverted action of the capillaries; and to restore a more efficient digestion and nutrition.

The first of these requires the use of anodynes, especially the preparations of opium; the second calls for the use of a class of remedies, the specific action of which is not easily described, but which are well known to the profession; such as nitrate of silver, sub-nitrate of bismuth, oil of turpentine, &c.; while the third is to be acccomplished chiefly by dietetic and hygienic regulations, sometimes aided by the preparations of iron. In the third case to which your attention has been called, we have endeavored to fulfil the several indications by giving the patient a powder of sub-nit. bismuth 10 grs., sulph. morph. $\frac{1}{8}$ of gr. every six hours, and three grains per. sulph. of iron in solution, between; while his diet has been chiefly boiled milk, slightly thickened with wheat flour.

He has already taken these remedies steadily for five or six days, resulting in so decided an improvement, that we shall continue it unchanged.*

For the treatment of the first class of cases, to which the first two patients belonged, we have the same indications with the addition of a fourth, namely: to use some alterative capable of inducing the re-absorption of those semiplastic infiltration or deposits which have caused the thickened and indurated condition of the affected portions of the mucous membrane. Here is one of the greatest difficulties in the treatment of this class of cases when they have become thoroughly chronic. The practitioner finds every new combination of anodynes and astringents that he may make, to relieve the patient *temporarily* only. The mercurials cannot be relied on for the desired alterative effect, because they increase the evacuations on the one hand, and diminish the plasticity of the already impoverished blood, on the other. While on a professional visit, recently, at the prisoners' camp on Rock Island, I was kindly shown through the hospitals by Drs. WATSON, GLEASON, and GILBERT.

* The two last mentioned cases both terminated fatally; one in forty-eight hours after its admission to the hospital, and the other in about five days. The third case, which in the above clinic was described as being treated on full doses of sub-nitrate of bismuth and sulphate morphine, alternated with per. sulphate of iron, was continued on the same treatment, only gradually lessening the quantity of morphine, for about three weeks, during which he fully recovered, and was discharged from the hospital.

Among them I found one devoted to diseases of the bowels, under the care of Dr. GILBERT, in which he had treated a considerable number of cases, both of diarrhœa and dysentery, with bromine held in solution by the aid of bromide of potassa, as the only remedy. Encouraged by his success, and hoping that the bromine might prove to be the alterative long needed in these cases, I commenced using it immediately on my return home. Thus far its effects have fully answered my expectations. The formulæ which I have used for adults is as follows:—

℞.—Bromine, ............ 16 gtts.
Bromide Potassa, ............ 20 grs.
Water, ............ ℥iv.

Mix, give a tea spoonful every 3 or 4 hours.

Although the two first cases to which I have called your attention, are already hopelessly exhausted, we will give them of this formulæ, a teaspoonful every two hours, and a powder of sub-nitrate of bismuth and morphine, each night and morning. We will also try to sustain the failing powers of life by milk-porridge and moderate doses of brandy and milk.

Their progress you will be able to determine in subsequent visits to the hospital wards.

